# A survey of multiple candidate probiotic bacteria reveals specificity in the ability to modify the effects of key wound pathogens

**DOI:** 10.1128/spectrum.00347-24

**Published:** 2024-05-03

**Authors:** Muna Alhubail, Andrew J. McBain, Catherine A. O'Neill

**Affiliations:** 1 Faculty of Biology, Medicine and Health, School of Biological Sciences, The University of Manchester, Manchester, United Kingdom; 2 Faculty of Biology, School of Health Sciences, Medicine and Health, The University of Manchester, Manchester, United Kingdom; Forschungszentrum Jülich GmbH, Juelich, Germany

**Keywords:** probiotic, keratinocyte, wound pathogen, lactic acid bacteria

## Abstract

We have evaluated the inhibitory effects of supernatants and lysates derived from several candidate probiotics, on the growth and biofilm formation of wound pathogens, and their ability to protect human primary epidermal keratinocytes from the toxic effects of pathogens. Supernatants (neutralized and non-neutralized) and lysates (*via* sonication) from *Lactiplantibacillus plantarum, Limosilactobacillus reuteri, Bifidobacterium longum, Lacticaseibacillus rhamnosus* GG, and *Escherichia coli* Nissle 1917 were tested for their inhibitory effects against *Staphylococcus aureus, Streptococcus pyogenes, Escherichia coli, Pseudomonas aeruginosa,* and *Acinetobacter baumanni*. The supernatants of *L. plantarum, L. rhamnosus, B. longum,* and *L. rhamnosus* GG reduced the growth of *S. aureus, E. coli,* and *A. baumanni. B. longum* additionally inhibited *P. aeruginosa* growth. However, neutralized *Lactobacillus* supernatants did not inhibit growth and in some cases were stimulatory. Lysates of *L. plantarum* and *L. reuteri* inhibited *S. pyogenes* while *B. longum* lysates inhibited *E. coli* and *S. aureus* growth. *E. coli* Nissle 1917 lysates enhanced the growth of *S. pyogenes* and *P. aeruginosa*. Biofilm formation by *E. coli* was reduced by lysates of *L. reuteri* and neutralized supernatants of all candidate probiotics. *P. aeruginosa* biofilm formation was reduced by *E. coli* Nissle supernatant but increased by *L. plantarum, L. reuteri,* and *Bifidobacterium longum* lysates. *L. reuteri* decreased the toxic effects of *S. aureus* on keratinocytes while *E. coli* Nissle 1917 lysates protected keratinocytes from *S. pyogenes* toxicity. In conclusion, lactobacilli and *E. coli* Nissle lysates confer inhibitory effects on pathogenic growth independently of acidification and may beneficially alter the outcome of interactions between host cell-pathogen in a species-specific manner.

**IMPORTANCE:**

One of the attributes of probiotics is their ability to inhibit pathogens. For this reason, many lactobacilli have been investigated for their effects as potential topical therapeutics against skin pathogens. However, this field is in its infancy. Even though probiotics are known to be safe when taken orally, the potential safety concerns when applied to potentially compromised skin are unknown. For this reason, we believe that extracts of probiotics will offer advantages over the use of live bacteria. In this study, we have surveyed five candidate probiotics, when used as extracts, in terms of their effects against common wound pathogens. Our data demonstrate that some probiotic extracts promote the growth of pathogens and highlight the need for careful selection of species and strains when probiotics are to be used topically.

## INTRODUCTION

Chronic wounds, such as diabetic foot ulcers and pressure sores, pose a significant burden to healthcare services which is likely to increase given the aging population (1, 2). While it is by no means clear what is cause and what is effect, a major factor determining whether a wound heals is the presence of infection (3, 4). Wound infection is usually caused by a consortium of microorganisms that can proliferate in the wound environment (5). Antibiotics play an important role in eliminating pathogens as they target central pathways regulating growth. However, their utility is likely to decrease in the future due to the development of resistance (6). This makes the requirement for new approaches to the management of wound infection urgent.

Probiotics have been demonstrated to have applications in the treatment and prevention, particularly of gastrointestinal infections (7–10). The various mechanisms employed include inhibition of pathogen adhesion to the epithelium and production of antimicrobial substances such as organic acids and bacteriocins (11–14). In recent years, interest in the use of probiotics has turned to epithelia other than the gut and the potential for these bacteria for the treatment of skin has started to gain research momentum (15–17). However, to date, most studies simply assess a single candidate probiotic and few authors have performed extensive comparison studies to match pathogens with the most efficacious probiotic.

Since probiotics are normally associated with the gut, there are potential safety concerns for their topical use especially on compromised skin. Although most probiotics are considered safe, their presence in the “wrong environment” could potentially induce bacteremia (18). One way to circumvent this potential problem is the use of bacterial extracts, often referred to as “postbiotics” (19). Previously, we have shown the potential of two so-called probiotics, *Limosilactobacillus reuteri* (*L. reuteri*) and *Lacticaseibacillus rhamnosus* GG, (*L. rhamnosus* GG) when used as lysates, to protect human primary keratinocytes from the toxic effects of the skin pathogen, *Staphylococcus aureus (S. aureus*). The mechanisms involved appeared to be due to the reduction in pathogen adhesion to the keratinocytes and was a function of the lysate of the organisms but not the supernatants (20–22). This suggests that it is not only the secreted components of probiotic bacteria that can be utilized as potential postbiotics. In the current study, we have extended our investigations to several more skin and wound-related pathogens: *Staphylococcus aureus*, *Streptococcus pyogenes*, *Escherichia coli*, *Pseudomonas aeruginosa,* and *Acinetobacter baumannii*. Here, we have chosen five candidate probiotics (*L. plantarum*, *L. reuteri*, *L. rhamnosus* GG, *Bifidobacterium longum,* and *E. coli* Nissle) and investigated the ability of their cell-free culture supernatants (including neutralized supernatants) or bacterial lysates, to inhibit wound pathogens in terms of growth and biofilm formation. Furthermore, we have assessed whether these candidate probiotics can protect human primary keratinocytes from the toxic effects of pathogenic bacteria.

## MATERIALS AND METHODS

### Bacterial strains

*L. rhamnosus* GG (ATCC 53103) and *Bifidobacterium longum* Reuter (ATCC BAA-999) were obtained from LGC Standard Limited (ATCC, Middlesex, UK). *L. plantarum* (ATCC 10241) was obtained from DSMZ (Leibniz Institute DSMZ, Germany) and *Acinetobacter baumannii* (NTC 12156) was obtained from National Collection of Type Culture operated by Public Health, England (NCTC, UK). *Escherichia coli* Nissle 1917 was isolated from Mutaflor tablets (Herdecke, Germany). *Staphylococcus aureus* WIBG 1.6, *Streptococcus pyogenes* WIBG 2.1, *Pseudomonas aeruginosa* WIBG 1.3, and *Escherichia coli* WIBG 2.4 were wild-type clinical stains isolated in the laboratory previously from a diabetic foot ulcer (23). *L. reuteri* (ATCC 55730) was the gift of Dr Gavin Humphreys, University of Manchester.

All bacteria were grown on Wilkins-Chalgren agar or broth. For *S. pyogenes*, Wilkins-Chalgren broth was supplemented with 5% horse serum (Sigma, UK).

*L. rhamnosus* GG, *L. reuteri*, *L. plantarum,* and *Bifidobacterium longum* were incubated anaerobically for 18–24 h at 37^°^C in a Mark 3 Anaerobic Workstation (Don Whitley Scientific, Shipley, UK), *Escherichia coli* Nissle 1917 and the pathogenic strains S. *aureus*, *S. pyogenes*, *P. aeruginosa*, *E. coli,* and *A. baumanni* were incubated aerobically for 18–24 h at 37^°^C in a static Memmert incubator (Memmert, GmBH, Germany).

### Preparation of bacterial supernatants and neutralized supernatants

10 mL stationary phase cultures of bacteria were harvested by centrifugation. The supernatant was then passed through a 0.22-µm pore size Millex-GV syringe filter (Millipore, Bedford, MA, USA) and divided into two 5 mL samples. The pH of the supernatant was measured using the Jenway 3510 pH meter (Fischer Scientific, UK). Supernatants were adjusted to 7.0 ± 0.2 using 1 M NaOH solution and then filtered. An equal volume of sterilized H_2_O was added to the non-neutralized supernatants to compensate for any dilution effects generated by the addition of NaOH.

### Preparation of bacterial lysates

Broth cultures (20 mL) of bacteria grown to the early stationary phase were harvested by centrifugation (1790rcf for 10 min). The cells were then washed twice in phosphate-buffered saline (PBS -pH 7.0) and the pelleted cells were resuspended in 2 mL PBS. The samples were then sonicated at 100% amplitude for 6–10 min on ice using a Bandelin Sonoplus sonicator (Bandelin, Berlin). Following sonication, the lysates were filtered using a 0.22-µm pore size Millex-GV (Bedford MA, USA) syringe filters. 50 µL of each lysate was spread, onto an agar plate and incubated overnight at 37°C to confirm the complete removal of all viable bacterial cells.

### Determination of inhibitory activity of lysates and supernatants on pathogenic growth

A stationary phase overnight broth culture of each pathogen was adjusted spectrophotometrically to a concentration of 10^6^ CFU/mL and a total of 200 µL (100 µL of the 10^6^ CFU/mL pathogen and 100 µL of lysate, supernatant, or neutralized supernatant) was inoculated into a 96-well plate. Control wells were inoculated with 100 µL of the 10^6^ CFU/mL of each pathogen together with either 100 µL of its lysate or 100 µL of its supernatant. The plate was then incubated in a Powerwave XS plate reader (Biotek, Bedfordshire, UK) at 37^°^C, where the absorbance of each well was measured at 660 nm every 1 h over 24 h. The growth curve of each organism was constructed and analyzed using the Gen5 Software program (Biotek, Bedfordshire, UK). The experiment was run in triplicate and repeated on at least three separate occasions for each organism.

### Testing the protective effect of lysates from candidate probiotics on the viability of human epidermal keratinocytes using flow cytometry

Primary normal human epidermal keratinocytes (NHEK) were purchased from Promocell (Germany) and grown and maintained as described previously (21). The cells were passaged into 12 well plates at a concentration of 5 × 10^4^ cells/mL.

The cells were then incubated with pathogens, bacterial lysates, or pathogens together with lysates. 24 h post-incubation, the viability of the cells was analyzed using an Annexin V apoptosis detection kit according to the manufacturer’s instructions (Thermo Fisher Scientific, UK) and DAPI (Thermo Fisher Scientific, UK). Briefly, after 24-h incubation, the media was removed from the cells and the wells were washed with phosphate buffer saline. 200 µL of keratinocyte trypsin was added to each well to detach the cells. Following detachment, 400 µL of trypsin inhibitor was added and cells were transferred to a 1.5 mL Eppendorf tube and centrifuged at 400 revolutions per minute (rpm) for 5 min. The supernatant was discarded and the pelleted cells were suspended in 200 µL 1X Annexin V binding buffer and re-centrifuged at 400 rpm for another 5 min. Again, after discarding the supernatant, the pellet was stained with 200 µL of diluted Annexin V antibody and kept on ice in the dark for 20 min. The cells were then centrifuged again at 400 rpm for another 5 min and the supernatant was discarded and the pellet was resuspended in 200 µL of the 1X Annexin V binding buffer containing 50 µL of 1 µg/mL of DAPI (4′,6, diamidino 2 phenylindole dihydrochloride). Cell viability analysis was performed using a CANTO-II flow cytometer. Cells that were negative for both Annexin V and DAPI were considered viable, Annexin V-positive cells were considered early apoptotic, and Annexin V and DAPI-positive cells were considered late apoptotic. DAPI-positive cells were considered to be necrotic. The data were analyzed using the Flow Jo software (FLOWJO, LLC). The experiment was repeated at least three times and run in triplicate for each organism.

### Assessment of biofilm formation

Biofilm formation was assessed using crystal violet staining of biofilms formed within MBEC (Minimum biofilm eradication concentration) Biofilm inoculators (Innovotech Inc, Edmonton, Canada). Briefly, 100 μL (10^6^ CFU/mL) of a culture of each pathogen grown in Wilkens-Chalgren broth was pipetted into the lower chamber of the MBEC device either with or without 100 μL of probiotic lysates or supernatants. Lysates and supernatants were added at the same time as pathogen in all experiments. 100 µL of each pathogens inoculated with 100 µL of its supernatant or lysate was used as a negative control. The samples were incubated for 72 h at 37°C after which they were washed in PBS and stained with 250 μL of 1% (vol/vol) crystal violet solution, destained with ethanol, and the absorbance at 590 nm read using a Powerwave XS plate reader (Biotek, Bedfordshire, UK). Each experiment was run with a minimum of *n* = 3 for each pathogen.

### Statistical analyses

Statistical analysis was performed using GraphPad Prism 7 software (obtained from http://www.graphpad.com). One-way ANOVA was used to analyze the significance of data generated for all experiments requiring comparison of the differences between more than two groups while student *t*-test was used to analyze the significant difference in experiments consisting of two groups. Each assay was performed in triplicate and repeated at least three times (*n* = 3) and the results were represented as the mean ± standard error of the mean (SEM). Significance was set at *P* < 0.05.

## RESULTS

### The inhibitory effect of probiotic supernatants is associated with acid production

The ability of supernatants from candidate probiotics to inhibit the planktonic growth of pathogens was assessed by co-incubating the filtered supernatant of each strain together with 10^6^ CFU/mL of each pathogen. The 10^6^ CFU/mL was chosen as it represents a physiologically relevant bacterial load in infected non-healing wounds (24). For the control, the 10^6^ CFU/mL of each pathogen was co-incubated with its filtered supernatant to maximize the equivalency in the nutritional status between the test and control. Some of the candidate probiotics are known to produce acid which could be inhibitory to pathogens. Therefore, to test for the effects of acid production (as opposed to other possible antimicrobial substances), pathogens were also incubated with a neutralized supernatant from each probiotic.

**Fig 1:**
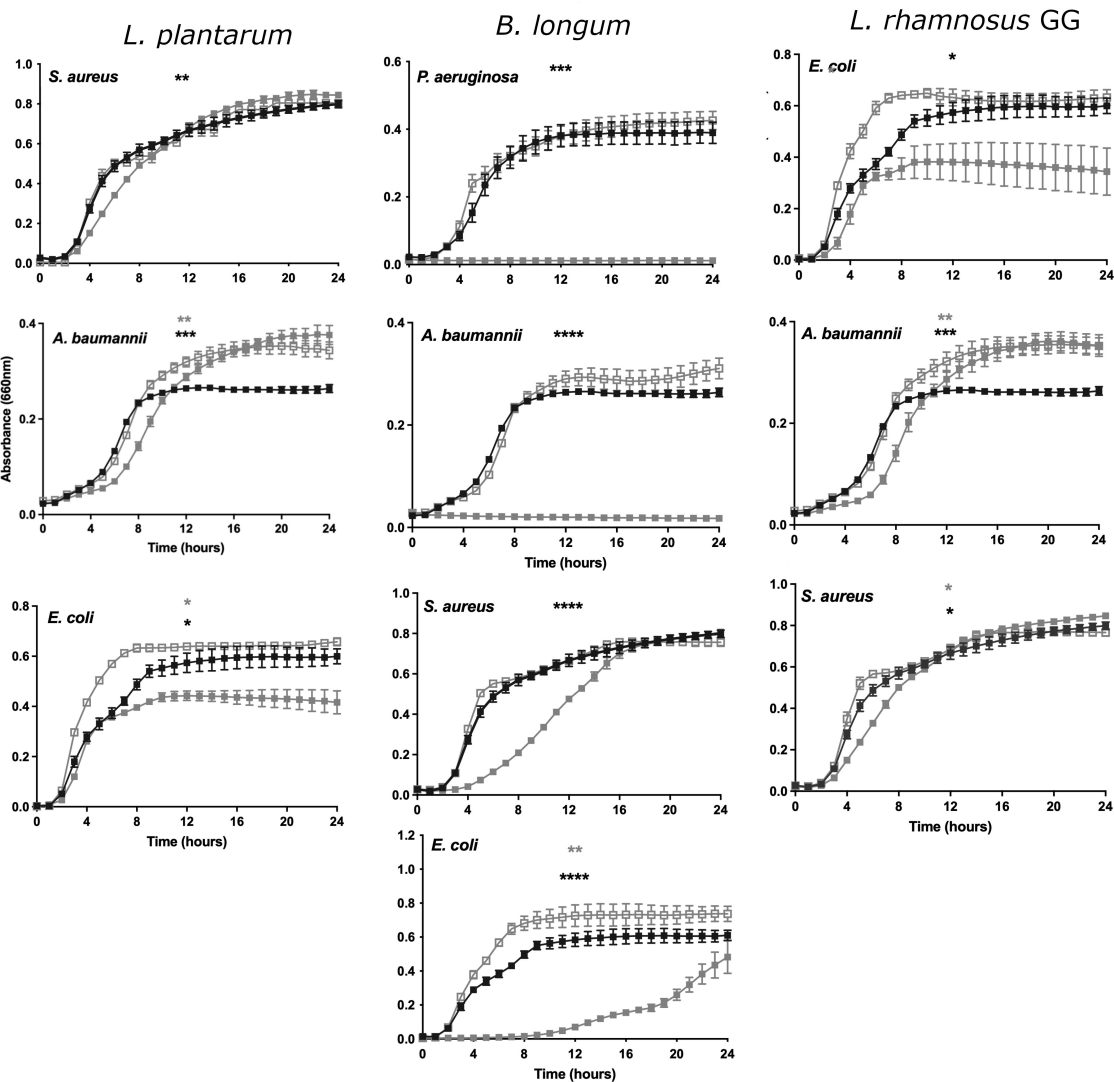
Supernatants from candidate probiotic bacteria affect the growth of specific pathogens. The black line shows the growth of the pathogen in the presence of its supernatant. The open gray line represents the growth of the pathogen with a neutralized supernatant. The closed gray line shows the growth of the pathogen in the presence of the probiotic supernatant.

The key data are shown in Fig. 1 with the data generated by all probiotic/pathogen combinations shown in Fig. S1.

The data in Fig. 1 show that the supernatants of *L. plantarum, B. longum,* and *L. rhamnosus GG* induced a significant reduction in the growth rate of *S. aureus*, *E.coli,* and *A. baumannii. B. longum* had an additional inhibitory effect against *P. aeruginosa* (Fig. 1). However, the use of a neutralized supernatant of the probiotics resulted in no inhibition of growth (Fig. 1), and in some cases, growth promotion (Fig. S1).

For *L. plantarum*, its inhibition of *S. aureus* and *A. baumanni* was characterized by a delay into the log phase of growth (Fig. 1 “*L. plantarum”*), whereas for *E. coli*, there was a reduction in absorbance at 24 h of around 20% (*P* < 0.05) in the presence of the supernatant compared to the absorbance without the supernatant (Fig. 1 “*L. plantarum*”).

The highest inhibitory effect of the *B. longum* supernatant was against both *P. aeruginosa* and *A. baumannii*, where the supernatant induced a significant reduction in absorbance at all stages of growth. At 24 h, the absorbance was 96.8% of that of the control (*P* < 0.0005) and 92.7% of that of the control (*P* < 0.0001), for *P. aeruginosa* and *A. baumannii,* respectively (Fig. 1 “*B. longum”*). For *E. coli*, the *B. longum* supernatant significantly inhibited the growth of the bacterium by 93.7%. However, at around 14 h, the inhibitory effect began to decline such that by 24 h, the absorbance in the treated sample was 38.4% of that in the control (*P* < 0.0001). For *S. aureus*, the supernatant of *B. longum* delayed the entry of the bacterium into the log phase and the absorbance of the treated sample was 47.6% (*P* < 0.0005) of that of the untreated at 10 h. However, as the bacterium entered the stationary phase, the inhibitory effect of the probiotic supernatant was lost and the absorbance value at 24 h was identical in treated and control samples (Fig. 1 “*B. longum”*).

The co-incubation of *L. rhamnosus* GG supernatant with wound-associated pathogens induced a significant reduction in the absorbance at 24 h of *E. coli* [Fig. 1 *“L. rhamnosus GG”* 33.9% (*P* < 0.05) of control value]. For *A. baumannii* (Fig. 1 *“L. rhamnosus GG”*) and *S. aureus* (Fig. 1 “*L. rhamnosus GG”*), the supernatant of *L. rhamnosus* GG induced a significant delay in the entry of the pathogen into log phase of growth (*P* < 0.005 and *P* < 0,05 respectively). However, *A. baumanii* remained in the log phase of growth for a longer time than the control before it entered the stationary phase resulting in significantly higher levels of bacteria at 24 h than in the control.

There was no significant inhibitory effect of any other probiotic supernatant tested against the pathogens, although some combinations resulted in the growth of pathogens when challenged with a neutralized supernatant (Fig. S1). Since the inhibitory effects were only observed with non-neutralized supernatants, the pH of the supernatants from an overnight growth of the probiotics was tested. The data in Table 1 show the pH of the supernatants. The supernatant of *B. longum* has the highest acidity followed by that of *L. rhamnosus* GG and *L. plantarum*. The data in Fig. 1 suggest that the organisms with the highest acidity also have the greatest inhibitory effects on growth, suggestive of the idea that the production of acid is the major mechanism underlying the effects on pathogenic growth.

**TABLE 1:** The pH of bacterial supernatants

Bacterium	pH of supernatant before neutralization
*L. plantarum*	4.79
*L. reuteri*	6.5
*B. longum*	4.57
*E. coli* Nissle 1917	6.6
*L. rhamnosus* GG	4.7

### Probiotic lysates are inhibitory to specific pathogens

The ability of bacterial lysates to inhibit the growth of different pathogens was assessed by co-incubating each lysate with 10^6^ CFU/mL of each pathogen in a 96-well plate. The growth rate was monitored for 24 h. For the control, the 10^6^ CFU/mL of each pathogen was co-incubated with its lysate to mimic the conditions in test wells and compensate for any dilution effects that could be caused by the addition of the lysate.

**Fig 2:**
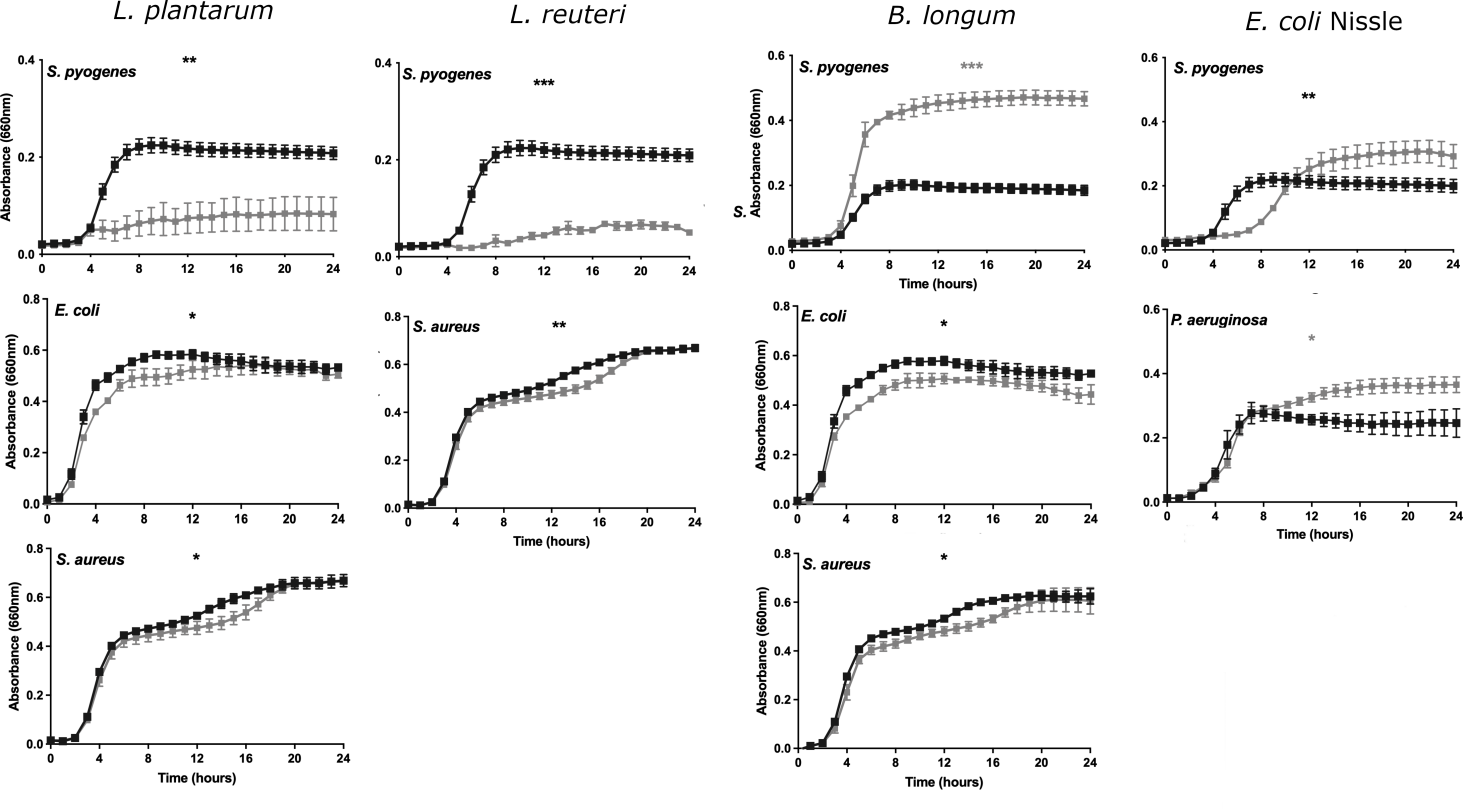
Lysates of probiotic bacteria have specific effects on pathogenic growth. The curves denote the growth of pathogens either in the presence of own lysate (black line) or lysates (gray line) of the candidate probiotics.

The *L. plantarum* lysate inhibited the growth of *S. pyogenes* such that at 24 h, there was a 68% reduction in the absorbance of the treated vs control well (*P* < 0.005, Fig. 2 *“L. plantarum”*). In addition, *L. plantarum* had a modest and transient inhibitory effect on the growth of *E. coli and S. aureus* (Fig. 2 *“L. plantarum”*).

The *L. reuteri* lysate was significantly inhibitory to the growth of *S. pyogenes* and at 24 h, the well containing treated cells had 80% less absorbance than control wells (*P* < 00005, Fig. 2 *“L reuteri”*). In addition, *L. reuteri* had a small transient effect on the growth of *S. aureus* which was apparent between 12 and 16 h. However, *L. reuteri* was not inhibitory to any other tested pathogen (Fig. 2 *“L. reuteri”*).

Inhibition of pathogens with the lysate derived from *B. longum* resulted in a significant increase in the growth of *S. pyogenes* (*P* < 0.0005) such that at 24 h, the absorbance of the treated well was more than double that of the untreated (Fig. 2 “*B. longum”*). By contrast, *B. longum* lysate was modestly inhibitory to the growth of *E. coli*, and transiently inhibitory to the growth of *S. aureus* (Fig. 2 *“B. longum”*).

The lysate of *E. coli* Nissle 1917 significantly delayed the entry of *S. pyogenes* into the log phase of growth. However, the pathogen reached a higher absorbance at 24 h in the presence of the lysate, than in its absence (Fig. 2 *“E. coli* Nissle”). The *E. coli* Nissle lysate also enhanced the growth of *P. aeruginosa* but had no effect on the growth of any other pathogens (Fig. 2 *“E. coli Nissle”*).

No other probiotic/pathogen combinations produced any effects on pathogenic growth rates (data not shown)

### Lysates from specific probiotics can protect epidermal keratinocytes from the toxic effects of pathogens

We tested the ability of the lysates shown to inhibit pathogenic growth, to protect human primary epidermal keratinocytes from the effects of pathogens. The specific combinations tested were as follows: *L. plantarum* vs *S. pyogenes, E. coli,* and *S. aureus;*

*L. reuteri* vs *S. aureus* and *S. pyogenes;* B. *longum* vs *S. aureus* and *E. coli; E. coli* Nissle vs *S. pyogenes*. These combinations were tested because of the observed inhibitory effects of these bacterial lysates against the specific pathogens noted above.

**Fig 3:**
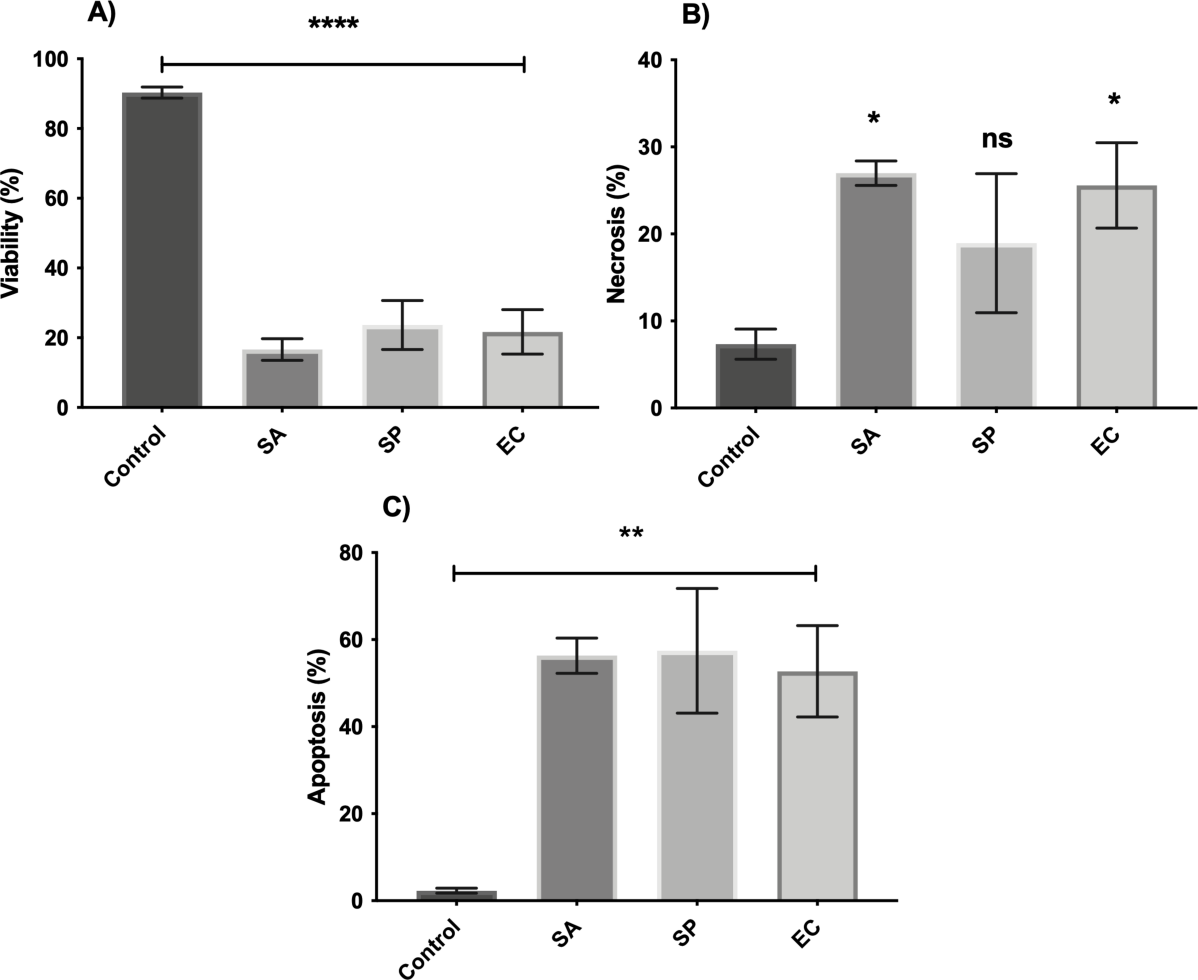
*S. pyogenes, S. aureus,* and *E. coli* induce significant cell death in human keratinocytes. Primary human keratinocytes were incubated with *S. pyogenes* (SP), *E. coli* (EC), or *S. aureus* (SA). The % viable cells (**A**), % necrotic cells (**B**), and % apoptotic cells (**C**) were measured by flow cytometry following 24-h incubation.

To begin with, the effects of the pathogen or lysates alone on the viability of human epidermal keratinocytes were investigated. All three pathogens (*S. aureus, S. pyogenes,* and *E. coli*) induced keratinocyte death, and only 15%–20% of the keratinocytes were viable following 24-h incubation with the pathogen (Fig. 3A). This appeared to be through a combination of increased apoptosis and necrosis (Fig. 3B and C). By contrast, none of the probiotic lysates (*L. plantarum, B. longum, E. coli* Nissle, and *L. reuteri*) induced any form of cell death in keratinocytes following 24-h incubation (Fig. S2).

**Fig 4:**
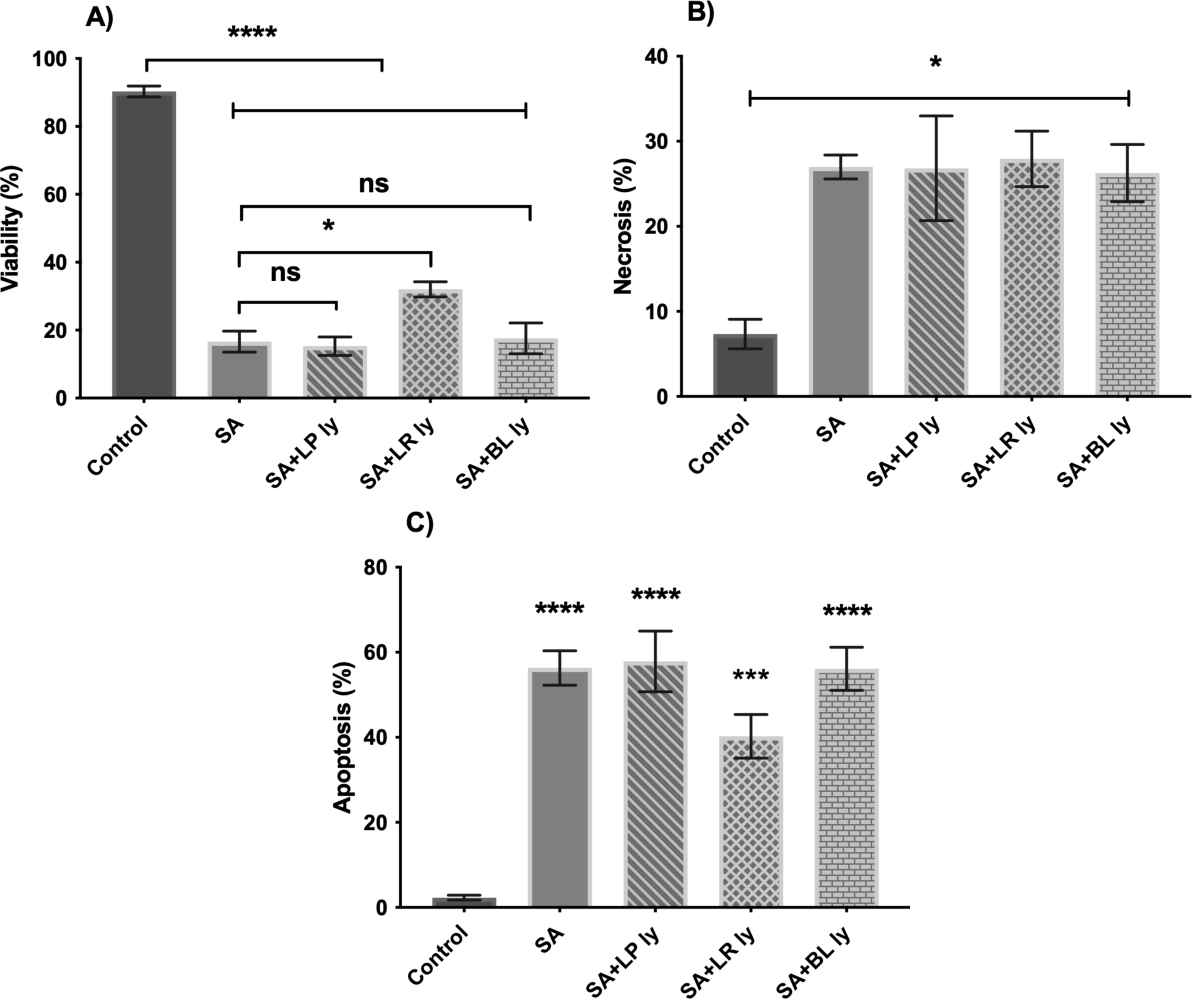
Lysates of *L reuteri*, but not *B. longum* or *L. plantarum* protect epidermal keratinocytes from the effects of *S. aureus*. Human epidermal keratinocytes were incubated with *S. aureus* (SA) either alone or in combination with lysates from *L. plantarum* (LP), *L. reuteri* (LR), or *B. longum* (BL). The % of viable cells (**A**), % necrotic cells (**B**), and five apoptotic cells (**C**) were measured at 24 h.

The toxic effects of *S. aureus* on keratinocytes were not mitigated by the presence of lysates from either *L. plantarum* or *B. longum*. However, there was an increase in the number of viable keratinocytes from 16% to 32% (*P* < 0.05) in the presence of the *L. reuteri* lysate (Fig. 4A through C).

**Fig 5:**
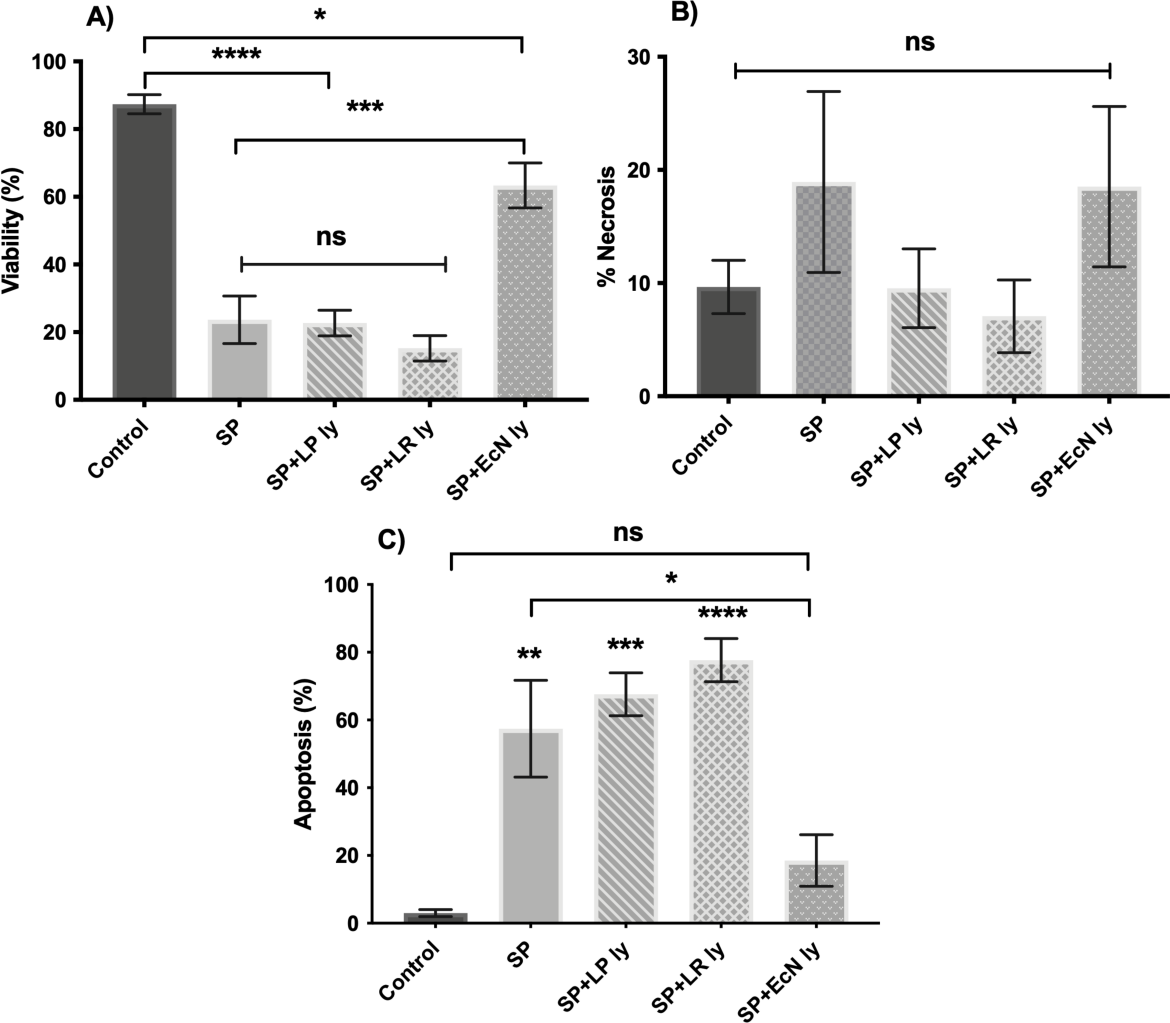
Lysates of *E. coli* Nissle, but not *L. plantarum* or *L. reuteri* protect epidermal keratinocytes from the effects of *S. pyogenes*. Human epidermal keratinocytes were incubated with *S. pyogenes* (SP) either alone or in combination with lysates (LY) from *L. plantarum* (LP) and *L. reuteri* (LR). The % of viable cells (**A**), % necrotic cells (**B**), and five apoptotic cells (**C**) were measured at 24 h.

The lysates of *L. plantarum* and *L. reuteri* could not protect epidermal keratinocytes from the effects of *S. pyogenes*. However, there was a significant increase in the number of viable keratinocytes when they were incubated with *S. pyogenes* in the presence of the *E. coli* Nissle lysate. In pathogen-treated keratinocytes, only around 20% remained viable following 24-h incubation. However, in the presence of the *E. coli* Nissle lysate, around 60% of keratinocytes were still viable following 24-h incubation (*P* < 0.0001). This appeared to be due to an inhibition of necrosis induced by the pathogen and there were no effects on apoptosis since *S. pyogenes* does not appear to induce keratinocyte apoptosis (Fig. 5A through C).

None of the lysates could protect keratinocytes from the toxic effects of *E. coli* (data not shown).

### Lysates and supernatants from specific probiotics inhibit biofilm formation by some pathogens

The ability of wound-associated pathogens to form a biofilm in the wound bed is considered one of the greatest challenges for wound management. Therefore, we investigated whether lysates or supernatants from any of the candidate probiotics could inhibit the biofilm-forming ability of wound pathogens.

**Fig 6:**
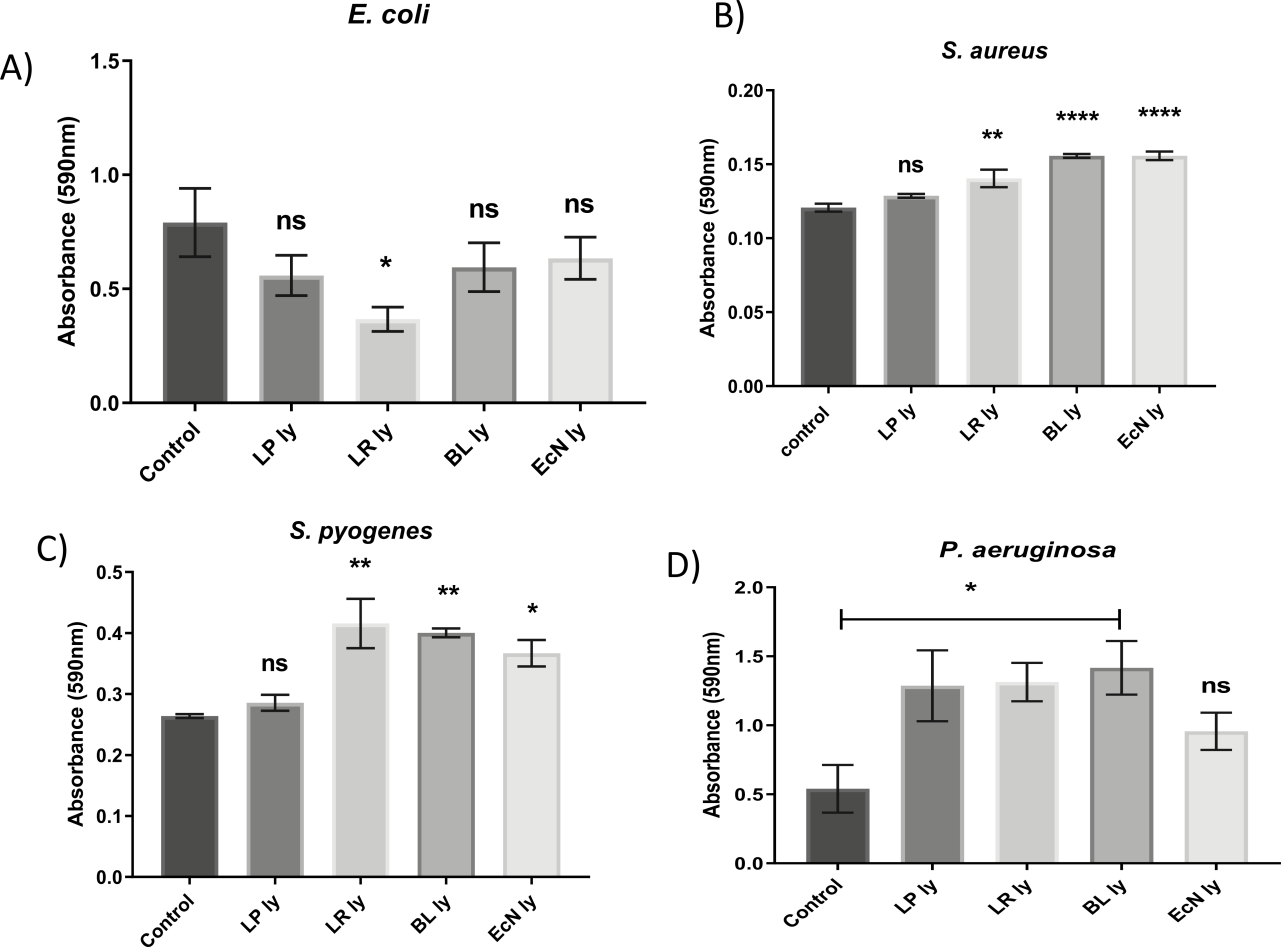
Lysates of specific candidate probiotics modify biofilm formation by wound pathogens. The co-incubation of lysates of *L. reuteri (LR*) with *E coli* (**A**) reduced biofilm formation. Probiotic lysates [*L. plantarum* (LP), B. *longum* (BL), *E coli* Nissle (ECN)] either had no effect or increased biofilm formed by *S. aureus* (**B**), *S. pyogenes* (**C**), and *P. aeruginosa* (**D**).

Except the *L. reuteri* lysates, which inhibited the formation of biofilms by *E. coli* by over 50% (Fig. 6A), none of the lysates tested could inhibit biofilm formation (data not shown). By contrast, biofilm formation by *S. aureus* (Fig. 6B)*, S. pyogenes* (Fig. 6C), and *P. aeruginosa* (Fig. 6D) was enhanced by the lysates of *L. reuteri* and *B. longum. E. coli* Nissle 1917 also increased the biofilm formed by *S. aureus* and *S. pyogenes* (Fig. 6B and C, respectively), whereas *L. plantarum* increased only the biofilm formed by *P. aeruginosa* (Fig. 6D).

**Fig 7:**
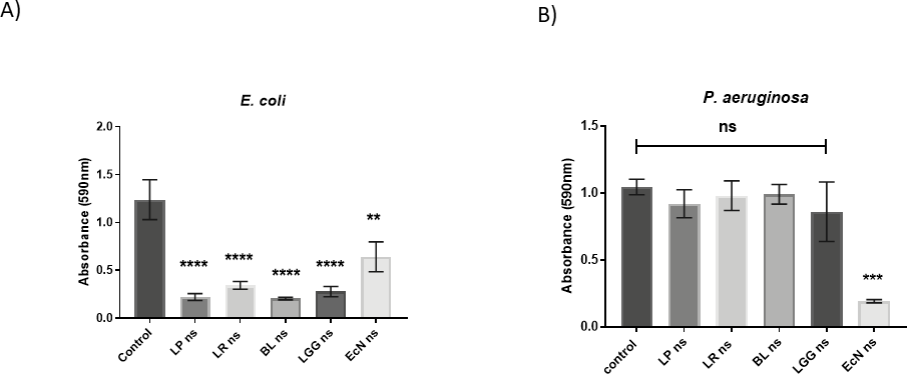
The supernatants from candidate probiotics reduce biofilm formation by wound pathogens in a selective manner. Neutralized supernatant (NS) from all test bacterial species (LP—*L. plantarum*, LR—*L. reuteri*, BL—*B. longum*, LGG—*L. rhamnosus* GG, ECN—*E. coli* Nissle) reduced biofilm formation by *E. coli* (**A**). Biofilm formation by *P. aeruginosa* was reduced only by the supernatant of *E. coli* Nissle 1917.

The use of a neutralized supernatant from candidate probiotic species resulted in a reduction in the formation of *E. coli* biofilm by all species (Fig. 7A). However, only the neutralized supernatant from *E. coli* Nissle 1917 inhibited *P. aeruginosa* biofilm formation (Fig. 7B). No other pathogen:supernatant combination resulted in changes to biofilm formation (data not shown).

## DISCUSSION

Wound infection impairs the healing process and prolongs the duration of wound closure (24). Several studies have examined the possibility of using topically applied probiotics in controlling and inhibiting wound infection (15, 16, 25, 26) with promising results. In the current investigation, we specifically investigated whether a range of lactic acid bacteria, a Bifidobacterium, and an *E. coli* Nissle 1917 could inhibit the growth of common wound-associated pathogens and protect keratinocytes from their toxic effects. Because even putatively safe bacteria such as lactobacilli have the potential to be harmful in an open wound, we tested only bacterial lysates and their cell-free supernatants since application in such forms is more likely to reach translation than live bacteria.

While the data generated here demonstrate that most supernatants are inhibitory to specific pathogens, the main mechanism underlying this appears to be the production of acid. This is evidenced by the observation that supernatants that are inhibitory lost their effect when the pH was increased to 7. Furthermore, there was an inverse relationship between the pH of the bacterial supernatant and its inhibitory effect.

The supernatant of *L. reuteri* was the only one with no inhibitory effect and since the *L. reuteri* supernatant was not acidic, this provides further evidence of the link between antimicrobial effects and acidity. *L. reuteri* is an obligate heterofermentative Lactobacillus (27, 28) and, as such utilizes the phosphogluconate pathway to ferment glucose into lactate, acetic acid, and CO_2_ in equimolar amounts. Homofermentative lactobacilli utilize the Embden-Meyerhof-Parnas (EMP) pathway to ferment glucose exclusively into lactic acid yielding 2 moles of lactic acid/1 mole of glucose (29, 30). As the concentration of the glucose in the medium used was not high (1 gm/L), this may explain why the *L. reuteri* supernatant did not require neutralization.

Putatively, probiotic lysates have the potential to inhibit wound-associated pathogens but in a highly species-specific manner Whilst none of the tested lysates could inhibit all pathogens, almost all the tested lysates were inhibitory to at least one pathogen. This was not due to any dilution effects through the addition of the lysates because all the experiments were controlled by the addition of a similar amount of the lysate prepared from the pathogen itself. However, of note is the observation that despite the inhibitory effect of *L. plantarum* and *B. longum* against some of the pathogens, both lysates resulted in a significant increase in the growth rate of specific pathogens. This suggests that these lysates might contain a potential nutrient source or other growth-promoting substances for pathogens. Therefore, this observation highlights the importance of careful selection of lysates for use against wound-associated pathogens.

A likely mechanism by which lysates inhibit the growth of specific pathogens is *via* the production of antimicrobial substances. However, acid is probably not responsible for the effects reported in the current study because the lysates were pH neutral. The production of the antimicrobial compounds Reuterin and Reutericyclin by strains of *L reuteri* has been previously reported (31). Reuterin is, however, produced only under conditions where glycerol is a carbon source (31); thus, the lack of glycerol in the media used to cultivate *L. reuteri* probably negates the possibility of reuterin production. However, the production of reutericyclin is a possibility and the known antimicrobial activity of this peptide is in keeping with the observed effects in this study (32). The antimicrobial substance in the *L. plantarum* lysate has activity against both Gram-positive and Gram-negative bacteria which is in keeping with reports of a broad spectrum of activity of “plantaricin” produced by some but not all strains of *L. plantarum* (33–35). The effect of *B. longum* lysate appeared to be selective, whereas, like *L. plantarum* lysate, although it showed an inhibitory effect against both Gram-positive and Gram-negative pathogens, this effect was restricted to a specific pathogen rather than all the tested pathogens from both categories. Several studies have also reported the production of antimicrobial substances with a broad range of activity by B. *longum*. However, the efficacy of these substances appears to be highly strain-specific (36–38). The lysate of *E. coli* Nissle 1917 was highly specific and affected only the growth of *S. pyogenes*. Several reports have demonstrated the inhibitory effect of *E. coli* Nissle 1917 on the growth of gut pathogens such as enterohemorrhagic *E. coli* (EHEC) strains (39–42). However, to date, its effectiveness against any wound-associated species has not been extensively studied.

A notable finding of this study is that the protective effects of putative probiotic lysates toward keratinocytes challenged with pathogens were not necessarily linked to antimicrobial activity. *L. reuteri* lysate did not protect keratinocytes from the toxic effects of *S. pyogenes* but was highly inhibitory to the growth of this pathogen. On the other hand*,* the lysate of *L. reuteri* provided a significant protective effect toward keratinocytes when co-incubated with *S. aureus* but was only modestly inhibitory to the growth of this pathogen. This result is in agreement with a previously reported finding by Prince et al. (20), who showed the co-incubation of *S. aureus* with either live culture of *L. reuteri* or its lysate-protected NHEKs (20). This appeared to be due to competitive exclusion for binding sites on keratinocytes rather than direct antimicrobial effects (20).

*E. coli* Nissle 1917 lysate provided significant protection to keratinocytes challenged with *S. pyogenes*. It was also the only lysate that induced a clear reduction in the apoptosis of NHEKs. *E. coli* Nissle 1917 lysate has antimicrobial effects against *S. pyogenes* as shown by the delayed entry of the pathogen into the log phase of the growth cycle. This could explain its protective effect on keratinocytes infected with this pathogen. However, the mechanism may also involve other effects such as interference with pathogen internalization which is a reported activity of *E. coli* Nissle against intestinal pathogens (43).

The use of lysates from *L. reuteri* specifically inhibited the production of biofilm from *E. coli*. This effect is probably not related to the inhibition of growth because *E. coli* growth was not inhibited by *L. reuteri* lysate. Similarly, the reduction in biofilm formed by *E. coli* in the presence of all probiotic supernatants and the anti-biofilm effects of *E. coli* Nissle against *P. aeruginosa* cannot be entirely explained in terms of growth reduction. However, our data are in keeping with several other studies where anti-biofilm effects have been observed independently of antimicrobial effects on planktonic growth (44, 45). In most studies, the effective molecules inhibited the motility of the organisms or the early stages of attachment and autoaggregation (46, 47). Indeed, further studies have shown that the supernatant of *E. coli* Nissle contains a proteinaceous factor that decreases motility and quorum sensing (48).

In summary, selected lysates or supernatants from candidate probiotics showed promise in early pre-clinical testing. This study however also emphasizes the importance of careful selection of LAB to maximize efficacy and to avoid any undesired enhancement of pathogenic growth or biofilm formation.

## Data Availability

The data underlying this article will be shared by reasonable request to the corresponding author.

## SUPPLEMENTAL MATERIAL

Supplementary material spectrum.00347-24-s0001.docx Fig. S1 and S2.

ASM does not own the copyrights to Supplemental Material that may be linked to, or accessed through, an article. The authors have granted ASM a non-exclusive, world-wide license to publish the Supplemental Material files. Please contact the corresponding author directly for reuse.
