# Supplementary material for: A survey of multiple candidate probiotic bacteria reveals specificity in the ability to modify the effects of key wound pathogens
Source: Microbiol Spectr. 2024 May 3;12(6):e00347-24. doi: 10.1128/spectrum.00347-24 (PMC11237428; doi:10.1128/spectrum.00347-24)

**S1** – The effects of bacterial supernatants on the growth of wound pathogens. The black line shows the growth of the pathogen in the presence of its own supernatant. The open gray line represents the growth of the pathogen with a neutralised supernatant. The closed gray line shows the growth of the pathogen in the presence of the LAB/*E. coli* Nissle supernatant.


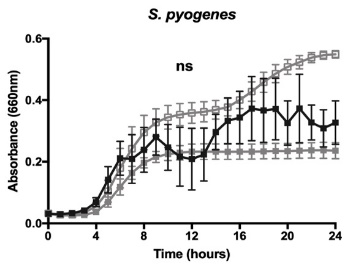

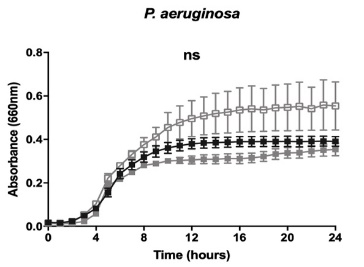


*L plantarum*


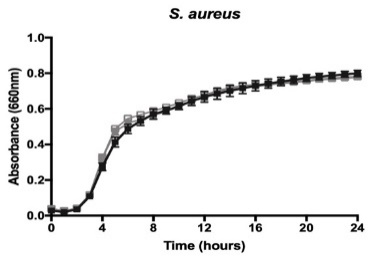

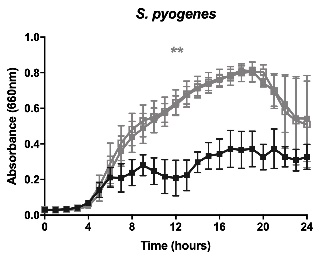

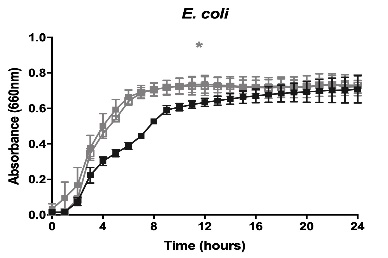

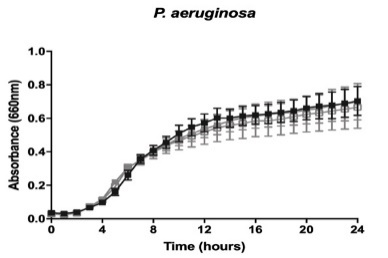

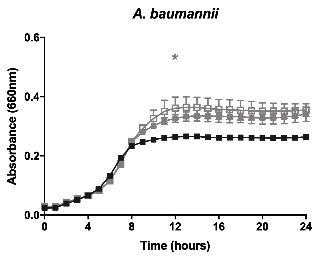

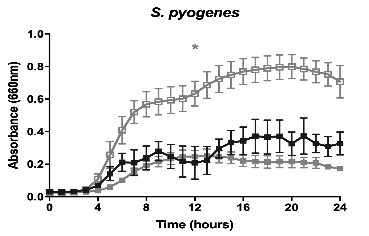


*B. longum*


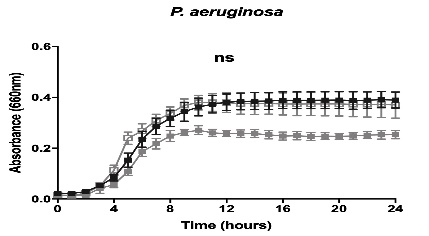

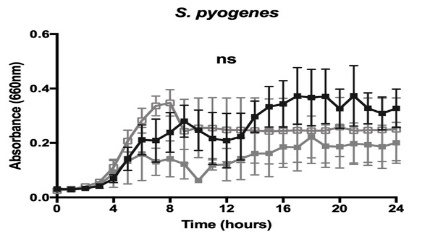


*L. rhamnosus GG*

*L. reuteri*


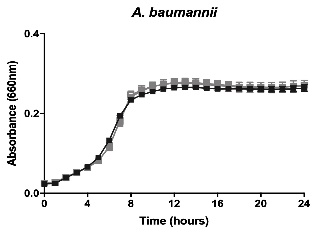

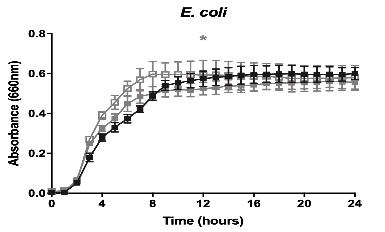

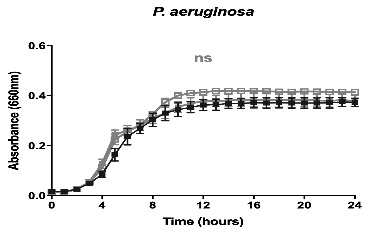

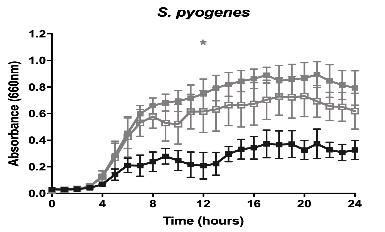

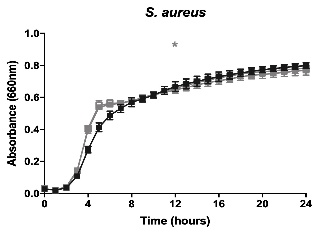


*E.coli* Nissle

**S2 -The effect of bacterial lysates on the viability of HEKs after 24 h incubation.** A) The viability of human epidermal keratinocytess was not affected after 24 h incubation with the lysates of *L. plantarum* (LP), *L. reuteri* (LR), *B. longum* (BL) and *E. coli* Nissle (EcN). B) No significant necrosis (B) or apoptosis (C) of keratinocytes was induced by any of the lysate. (n=3) where n represents the number of biological replicates. Error bars represent ± standard error of the mean (SEM). Ns denotes non-significant data


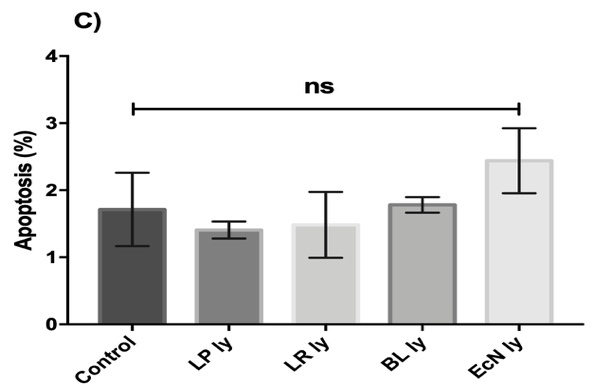

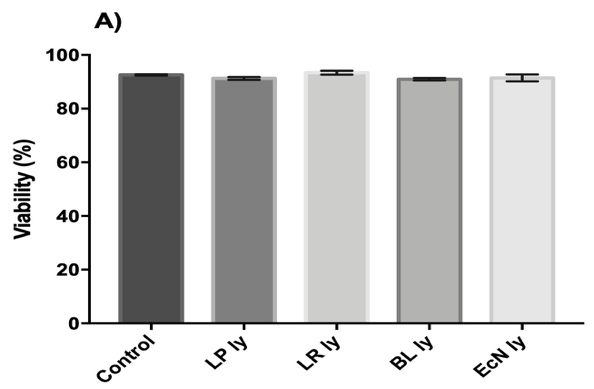

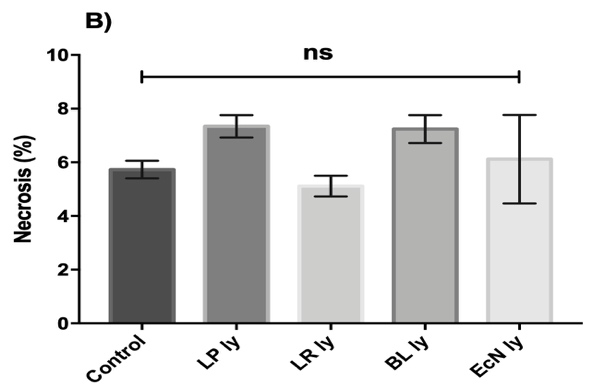

Supplement: Supplementary material — Fig. S1 and S2. [file spectrum.00347-24-s0001.docx]
